# A highly nonlinear S-box based on a fractional linear transformation

**DOI:** 10.1186/s40064-016-3298-7

**Published:** 2016-09-26

**Authors:** Shabieh Farwa, Tariq Shah, Lubna Idrees

**Affiliations:** 1 Department of Mathematics, COMSATS Institute of Information Technology, Wah Cantt, Pakistan; 2 Department of Mathematics, Quaid-i-Azam University, Islamabad, Pakistan

**Keywords:** S-box, Galois field, Fractional linear transformation, Majority logic criterion

## Abstract

We study the structure of an S-box based on a fractional linear transformation applied on the Galois field $GF(2^{8})$. The algorithm followed is very simple and yields an S-box with a very high ability to create confusion in the data. The cryptographic strength of the new S-box is critically analyzed by studying the properties of S-box such as nonlinearity, strict avalanche, bit independence, linear approximation probability and differential approximation probability. We also apply majority logic criterion to determine the effectiveness of our proposed S-box in image encryption applications.

## Background

The advanced encryption standard (AES) (Daemen and Rijmen 2002) is based on the substitution permutation network (SPN) which applies several layers of substitution and permutation. In any SPN, substitution followed by permutation is performed certain number of times to encrypt the plaintext into ciphertext in order to assure secure communication (Daemen and Rijmen 2002). The choice of a substitution box (S-box) (Shannon 1949) is the most sensitive step in determining the strength of a cryptosystem against several attacks. It is therefore essential to understand the design and properties of an S-box for encryption applications (Detombe and Tavares 1992). The improved quality of the S-Box to enhance the confusion creating capability in certain SPN has been a challenge for researchers.

In literature many algorithms for algebraically complex and cryptographically strong S-boxes, such as AES, APA, Gray, Skipjack, Xyi and Residue Prime (RP) S-boxes, are available. For the interest of readers we give a brief description of these structures. The AES S-box is based on the composition of inversion map and the affine transformation. It is a non-Feistel cipher. The algebraic expression of AES S-box is simple and involves only nine items (Daemen and Rijmen 2002). The structure of APA S-box uses composition of affine surjection, power function and again affine surjection. This design improves the algebraic complexity from 9 to 253 as compared to the AES S-box (Cui and Cao 2007). The Gray S-box is obtained from the AES S-box with an additional transform based on binary Gray codes. It inherits all the important cryptographic properties of AES S-box with an increased security against attacks (Tran et al. 2008). Skipjack is a Feistel network based on 32 rounds. This algorithm uses an 80-bit key to encrypt or decrypt 64-bit data blocks. The S-box based on Skipjack algorithm is also known as Skipjack F-table (Kim and Phan 2009). The XYi S-box is a mini version of a block cipher with block size of 8 bits. It has increased efficiency in computer applications (Shi et al. 2002). The Residue Prime S-box uses the field of residues of a prime number as an alternative to the Galois field based S-boxes (Abuelyman and Alsehibani 2008). These widely used S-boxes play the role of benchmarks in the field of cryptography. Among these, AES, APA and Gray S-boxes attain the highest nonlinearity measure 112. The S-box algorithm proposed in this framework produces high nonlinearity effect as achieved by the top S-boxes AES, APA and Gray, however, unlike these S-boxes, our S-box is structured by employing a single direct map rather the composition of two or more maps which makes this algorithm more efficient and economic in both software and hardware applications.

It is highly desired property for a cryptographically strong S-box to show good resistance towards linear and differential cryptanalysis (Biham and Shamir 1991; Matsui 1998). For a Boolean function *f*, the linear cryptanalysis is based on finding affine approximation to the action of a cipher (Nyberg 1993). Recently some efficient models are studied for S-boxes based on fractional linear transformations (Hussain et al. 2011, 2013a, b). S-box being the only nonlinear component in block cipher always requires high nonlinearity effect (Carlet and Ding 2004, 2007; Nyberg 1992, 1993). Motivated by some recently presented designs, we in this paper propose an algorithm to structure an $8 \times 8$ S-box using fractional linear transformation applied on the Galois field $GF(2^{8})$ which produces very high nonlinearity measure. We further analyse the properties of the new S-box by different commonly used tests such as nonlinearity, strict avalanche criterion (SAC), bit independent criterion (BIC), linear and differential approximation probability tests (LAPT, DAPT). We then compare the results with those for the famous S-boxes and observe that our new S-box, based on a simple and straightforward algorithm, produces coherent results.

The material presented in this paper is organized as follows. In “Algorithm for S-box” section we explain in detail the construction and major properties of the underlying Galois field $GF(2^{8})$. We further discuss some interesting features of the fractional linear transformation and describe how this transformation is applied on the Galois field to structure the new S-box. “Analyses of S-box” section deals with the analysis of S-box against several common attacks and compares the cryptographic potential of our proposed S-box with other S-boxes such as AES, APA, Gray, Skipjack, Xyi and Residue Prime. In “Statistical analyses of S-box” section we perform some statistical analysis based on the image encryption application of the S-box and in “Conclusion” section we present conclusion regarding the significance of the new S-box when critically observed in comparison with the previously known models.

## Algorithm for S-box

This section mainly deals with the structure of our S-box. Before we discuss the constituent algorithm, we need to go through some fundamental facts.

A function $f: {\mathbb {F}}_{2}^{n}\rightarrow {\mathbb {F}}_{2}$ is called a *Boolean function*. We define a *vectorial Boolean function*$F: {\mathbb {F}}_{2}^{n}\rightarrow {\mathbb {F}}_{2}^{m}$ as

$$\begin{aligned} F(x)=(f_{1}(x),\,f_{2}(x),\ldots ,f_{m}(x)), \end{aligned}$$

where $x=(x_{1},\, x_{2},\ldots ,x_{n})\in {\mathbb {F}}_{2}^{n}$ and each of $f_{i}$’s for $1\le i\le m$ is a Boolean function referred to as coordinate Boolean function. An $n \times n$ S-box is precisely defined as a vectorial Boolean function $S: {\mathbb {F}}_{2}^{n}\rightarrow {\mathbb {F}}_{2}^{n}$.

At this stage, it seems quite practical to understand the structural properties of the Galois field used to construct an S-box. Generally for any prime *p*, Galois field $GF(p^{n})$ is given by the factor ring ${\mathbb {F}}_{p}[X]/ <\eta (x)>$ where $\eta (x)\in {\mathbb {F}}_{p}[X]$ is an irreducible polynomial of degree *n*.

For an $8 \times 8$ S-box, we use $GF(2^{8})$. In advanced encryption standards (AES), the construction of $GF(2^{8})$ is based on the degree 8 irreducible polynomial $\eta (x)=x^{8}+x^{4}+x^{3}+x+1$. In Hussain et al. (2013b), $\eta (x)=x^{8}+x^{4}+x^{3}+x^{2}+x+1$ is used as the generating polynomial. Here we choose $\eta (x)=x^{8}+x^{6}+x^{5}+x^{4}+1$ as the irreducible polynomial that generates the maximal ideal $<\eta (x)>$ of the principal ideal domain ${\mathbb {F}}_{2}[X]$. It is important to note that we may choose any degree 8 irreducible polynomial for constructing $GF(2^{8})$ however the choice of generating polynomial may affect our calculations as the binary operations are carried modulo the used polynomial (see Benvenuto 2012 for details).

Generally the construction of an S-box requires a nonlinear bijective map. In literature many algorithms based on such maps or their compositions are presented to synthesize cryptographically strong S-boxes. We present the construction of S-box based on an invertible nonlinear map known as the fractional linear transformation. It is a function of the form $\frac{az+b}{cz+d}$ generally defined on the complex plain ${\mathbb {C}}$ such that *a*, *b*, *c* and $d \in {\mathbb {C}}$ satisfy the non-degeneracy condition $ad-bc\ne 0$. The set of these transformations forms a group under the composition. The identity element in this group is the identity map and the the inverse $\frac{dz-b}{-cz+a}$ of $\frac{az+b}{cz+d}$ is assured by the condition $ad-bc\ne 0$. One can easily observe that the algebraic expression of this map has a combined effect of inversion, dilation, rotation and translation. The nonlinearity and algebraic complexity of the fractional linear transformation motivates the idea to employ this map for byte substitution.

For the proposed S-box we apply fractional linear transformation *g* on the Galois field discussed above, i.e. $g:GF(2^{8})\rightarrow GF(2^{8})$ given by $g(t)=\frac{at+b}{ct+d}$, where $a,\, b,\, c$ and $d\in GF(2^{8})$ such that $ad-bc\ne 0$ and *t* varies from 0 to $255 \in GF(2^{8})$. We may choose any values for parameters *a*, *b*, *c* and *d* that satisfy the condition $ad-bc\ne 0$. Here, for calculations, we take $a=29=00011101,\, b=15=00001111,\,c=8=00001000$ and $d=9=00001001$. One may observe that as we are working on a finite field, *g*(*t*) needs to be explicitly defined at $t=47$ (at which denominator vanishes), so in order to keep *g* bijective we may define the transformation as given below;

$$\begin{aligned} g(t): {\left\{ \begin{array}{ll} \frac{29t+15}{8t+9};&{}\quad t\ne 47\\ 149;&{}\quad t=47 \end{array}\right. } \end{aligned}$$

Following the binary operations defined on the Galois field (Benvenuto 2012), we calculate the images of *g* as shown in Table 1.

**Table 1:** Images of *g*

$t \in {\mathbb {Z}}_{2^{8}}$	$t\in GF(2^{8})$	*g*(*t*)
0	00000000	124
1	00000001	18
.	.	.
.	.	.
.	.	.
255	11111111	138

Thus the images of the above defined transformation yield the elements of the proposed S-box (see Table 2).

**Table 2:** S-box

124	18	154	77	3	216	99	81	117	91	112	125	88	32	10	96
227	253	141	194	235	5	111	9	122	37	206	233	156	72	53	51
184	7	20	239	102	22	166	210	192	97	226	27	12	248	79	149
69	59	196	220	132	109	94	168	234	84	15	108	120	52	142	14
25	90	151	205	93	0	26	171	217	41	1	67	224	197	21	198
130	174	231	161	199	153	76	6	144	170	246	221	43	232	29	219
61	229	191	242	195	95	137	225	157	75	39	119	44	98	104	87
115	89	56	110	160	42	31	249	169	222	146	11	245	238	136	247
54	139	200	8	36	46	126	218	121	165	105	16	58	35	135	164
207	230	2	243	63	123	214	80	68	55	183	114	107	208	62	163
252	145	116	250	13	204	127	228	187	113	49	86	159	83	152	244
180	193	57	173	133	128	150	30	40	190	255	240	237	155	85	175
162	47	134	50	60	28	186	177	33	202	176	19	70	209	24	178
71	38	212	48	201	172	129	143	215	188	181	147	158	65	101	100
251	179	182	203	140	223	66	254	64	23	45	189	17	213	131	4
73	211	167	74	78	148	236	185	92	241	82	103	118	106	34	138

It is important to mention that an $8 \times 8$ S-box has 8 constituent Boolean functions. A Boolean function *f* is *balanced* if $\{x|f(x)=0\}$ and $\{x|f(x)=1\}$ have same cardinality or the Hamming weight HW$(f)=2^{n-1}$. The significance of the balance property is that the higher the magnitude of a function’s imbalance, the more likelihood of a high probability linear approximation being obtained. Thus, the imbalance makes a Boolean function weak in terms of linear cryptanalysis. Furthermore, a function with a large imbalance can easily be approximated by a constant function. All the Boolean functions $f_{i},\,i \le i \le 8$, involved in the S-box as shown in Table 2 satisfy the balance property. Hence, the proposed S-box is balanced. It might be of interest that in order to choose feasible parameters leading to balanced S-boxes satisfying all other desirable properties (as discussed in the next section), one can use *constraint programming*, a problem solving strategy which characterises the problem as a set of constraints over a set of variables (Kellen 2014; Ramamoorthy et al. 2011).

An S-box is used to convert the plain data into the encrypted data, it is therefore essential to investigate the comparative performance of the S-box. We, in the next section, analyse the newly designed S-box through various indices to establish the forte of our proposed S-box.

## Analyses of S-box

For the assessment of the cryptographic strength of our S-box, in this section, we apply some widely used analysis techniques such as nonlinearity, bit independence, strict avalanche, linear and differential approximation probabilities etc. In the following subsections we present all these performance indices one by one.

### Nonlinearity

The nonlinearity indicator counts the number of bits which must be altered in the truth table of a Boolean function to approach the nearest affine function.

**Fig. 1:**
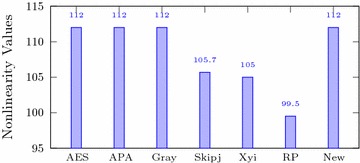
Nonlinearity of different S-boxes

 Table 3 shows that for the newly designed S-box, the average nonlinearity measure is 112. Figure 1 shows that when we compare this with different famous S-boxes, the nonlinearity of the proposed S-box is similar to that of the top S-boxes such as AES, APA and Gray and much higher then that of the Skipjack, Xyi and Residue Prime S-boxes.

**Table 3:** Performance Indices for new S-box

Analysis	Max.	Min.	Average	Square deviation	The differential approximation probability	The linear approximation probability
Nonlinearity	112	112	*112*			
SAC	0.5625	0.453125	*0.510254*	0.0165278		
BIC		112	*112*	0		
DP					*0.015625*	
LP	144					*0.0625*

Italic values are used for comparison purposes

**Table 4:** Comparison of performance indices for different S-boxes

S-box	Nonlinearity	SAC	BIC	DP	LP
AES	112	0.5058	112.0	0.0156	0.062
APA	112	0.4987	112.0	0.0156	0.062
Gray	112	0.5058	112.0	0.0156	0.062
Skipjack	105.7	0.4980	104.1	0.0468	0.109
Xyi	105	0.5048	103.7	0.0468	0.156
RP	99.5	0.5012	101.7	0.2810	0.132
*New*	*112*	*0.510254*	*112*	*0.015625*	*0.0625*

Italic values are used for comparison purposes

### Linear approximation probability

The linear approximation probability determines the maximum value of imbalance in the event. Let $\Gamma _{x}$ and $\Gamma _{y}$ be the input and output masks respectively and *X* consists of all possible inputs with cardinality $2^{n}$, the linear approximation probability for a given S-box is defined as;

$$\begin{aligned} LP=\underset{\Gamma _{x},\Gamma _{y}\ne 0}{\max }\left| \frac{ \#\{x|x.\Gamma _{x}=S(x).\Gamma _{y}\}}{2^{n}}-\frac{1}{2}\right| \end{aligned}$$

**Fig. 2:**
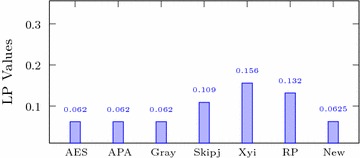
LP of different S-boxes

Table 4 and Fig. 2 show that the linear approximation probability of the newly structured S-box is much better than those for Skipjack, Xyi and Residue prime S-boxes.

### Differential approximation probability

**Fig. 3:**
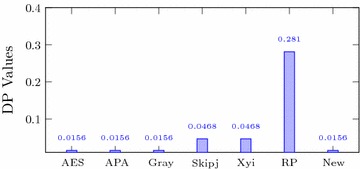
DP of different S-boxes

The differential approximation probability is defined as;

$$\begin{aligned} DP=\left[ \frac{\#\{x\in X|S(x)\oplus S(x\oplus \Delta x)=\Delta y\}}{2^{n}}\right] , \end{aligned}$$

where $\Delta x$ and $\Delta y$ are input and output differentials respectively. In ideal conditions, the S-box shows differential uniformity (Biham and Shamir 1991). The smaller the differential uniformity, the stronger is the S-box. It is evident from the Table 4 and Fig. 3 that the differential approximation probability of the proposed S-box is similar to those of the AES, APA and Gray S-boxes and much better than the Skipjack, Xyi and Residue Prime S-boxes.

### Strict avalanche criterion

**Fig. 4:**
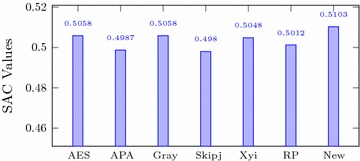
SAC of different S-boxes

For any cryptographic design, when we change the input bits, the performance of the output bits is examined by this criterion. It is desired that a change in a single input bit must cause changes in half of the output bits. In other words a function $F:{\mathbb {F}}_{2}^{n}\rightarrow {\mathbb {F}}_{2}^{n}$ is said to satisfy SAC if for a change in an input bit $i \in \{1,\,2, \ldots ,n\}$ the probability of change in the output bit $j \in \{1,\,2, \ldots , n\}$ is 1/2. It is clear from the results shown in Table 4 and Fig. 4 that our S-box satisfies the requirements of this criterion.

### Bit independence criterion

The criterion of bit independence, introduced by Webster and Tavares (1986), is used to analyse the behaviour of bit patterns at the output and the effects of these changes in the subsequent rounds of encryption (Tran et al. 2008). For any vector Boolean function $F:{\mathbb {F}}_{2}^{n}\rightarrow {\mathbb {F}}_{2}^{n}$, $\forall \, i,j$ and $k\in \{1,\,2,\ldots ,\,n\}$ with $j\ne k$, inverting input bit *i* causes output bits *j* and *k* to change independently. In cryptographic systems it is highly desired to increase independence between bits as it makes harder to understand and forecast the design of the system.

**Fig. 5:**
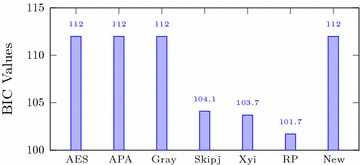
BIC of different S-boxes

The numerical results of BIC are given in Table 4 and are compared in Fig. 5. It can be observed that according to these results our S-box is quite similar to the AES, APA and Gray S-boxes.

**Table 5:** Comparison of MLC for new S-box and different S-boxes

Images	Entropy	Contrast	Correlation	Energy	Homog.
Plain image	7.4451	0.2100	0.9444	0.1455	0.9084
AES	7.2531	7.5509	0.0554	0.0202	0.4662
APA	7.2264	8.1195	0.1473	0.0183	0.4676
Gray	7.2301	7.5283	0.0586	0.0203	0.4623
Skipjack	7.2214	7.7058	0.1025	0.0193	0.4689
Xyi	7.2207	8.3108	0.0417	0.0196	0.4533
RP	7.2035	7.6236	0.0855	0.0202	0.4640
*New*	*7.2415*	*7.4568*	*0.0785*	*0.0223*	*0.4731*

Italic values are used for comparison purposes

## Statistical analyses of S-box

In this section we present some useful statistical analysis of the new and some famous S-boxes. We apply the majority logic criterion (Hussain et al. 2012) in order to determine the effectiveness of the proposed S-box in image encryption applications.

Due to the expeditious developments in the area of digital image processing, it is quite challenging to protect the digital information against different attacks. In the last few years many efficient algorithm have been presented by the researchers regarding secure image encryption schemes (Bao and Zhou 2015; Gao and Chen 2008; Murguia et al. 2012; Ramirez-Torres et al. 2014; Vargas-Olmos et al. 2015, 2016). During the image encryption process distortions occur and the strength of the encryption algorithm used is characterized by the type of these distortions. We examine this by using various parameters generated by different statistical analysis regarding entropy, contrast, correlation, energy and homogeneity respectively. We begin with the entropy analysis which is used to measure the randomness in a system. This characterizes the texture of image. Some other analyses (as named above) are also applied in combination with the entropy analysis to enhance the authenticity of the results regarding the performance of an S-box. Contrast analysis measures the ability to identify objects in an image. To ensure strong encryption an elevated level of contrast is required. Correlation analysis is used to analyze the statistical properties of an S-box. By this analysis we determine the similarity between the pixels patterns of the plain and the encrypted images. Energy analysis determines the measure of the energy of an encrypted image when processed by various S-boxes. This measure gives the sum of squared elements in GLCM. The homogeneity analysis is used to determine the closeness of the elements distribution in the grey level co-occurrence matrix (GLCM) to GLCM diagonal. It is worth mentioning that a strong encryption algorithm requires a small measure of correlation, energy and homogeneity however high value of entropy and contrast.

**Table 6:** Results for encryption using new S-box in different noisy environments

Images	Entropy	Contrast	Correlation	Energy	Homog.
Plain image	7.4451	0.2100	0.9444	0.1455	0.9084
Encryption at $\sigma =25$	7.1936	7.3974	0.0068	0.0153	0.3934
Encryption at $\sigma =50$	7.1545	7.2851	0.0043	0.0161	0.3992
Encryption at $\sigma =75$	7.1269	6.5029	0.0015	0.0180	0.4133

**Table 7:** Results for encryption using AES S-box in different noisy environments

Images	Entropy	Contrast	Correlation	Energy	Homog.
Encryption at $\sigma =25$	7.1438	7.4417	0.0047	0.0159	0.3921
Encryption at $\sigma =50$	7.0705	7.3104	0.0033	0.0162	0.3933
Encryption at $\sigma =75$	7.0475	7.124	0.0017	0.0178	0.4023

**Fig. 6:**
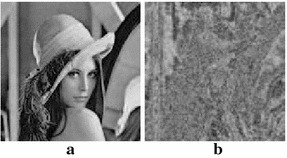
Lena’s plain image and its encryption using New S-box. **a** Plain image. **b** Encrypted Image

**Fig. 7:**
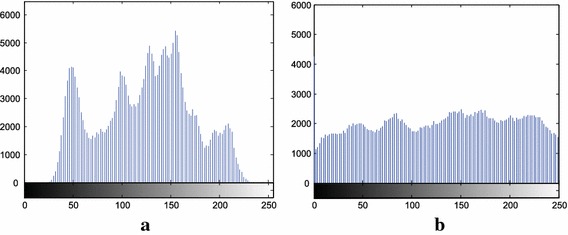
Histogram of the images in Fig. 6. **a** Plain image. **b** Encrypted Image

Figure 6 shows the plain image of Lena and its encryption using the new S-box. It is quite obvious from the visual results that our method of encryption creates acceptable level of confusion.

For an image, its histogram graphically represents image-pixels distribution by plotting the number of pixels at each intensity level (Ramirez-Torres et al. 2014). It has been established that the histogram of the original and the encrypted image should be significantly different so that attackers could not extract the original image from the encrypted one. Figure 7 shows the respective histograms of Lena’s plain image and its encrypted version. The histogram analysis evidently proves the stability of our proposed method against any histogram based attacks.

In order to determine the quantitative measure of the efficiency of the proposed method in image encryption, MLC is applied on a typical $512 \times 512$ image of Lena for the new S-box and results are compared with the other famous S-boxes. The numerical results for correlation, entropy, contrast, homogeneity and energy are arranged in Table 5. It is observed that the proposed S-box satisfies all the criteria to be used for the safe communication.

**Fig. 8:**
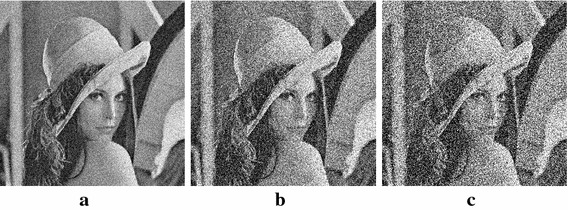
Noise-effected images. **a** $\sigma =25$, **b** $\sigma =50$, **c** $\sigma =75$

**Fig. 9:**
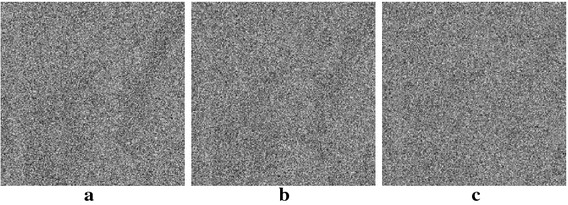
Encryption with the proposed S-box in noisy environments. **a** Encryption of Fig. 8a. **b** Encryption of Fig. 8b. **c** Encryption of Fig. 8c

**Fig. 10:**
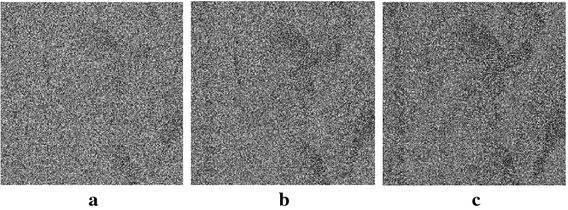
Encryption with the AES S-box in noisy environments. **a** Encryption of Fig. 8a. **b** Encryption of Fig. 8b. **c** Encryption of Fig. 8c

We may further test the performance of the proposed method in noisy environments. For this purpose, we consider $\Omega \subset {\mathbb {Z}}^2$ as a bounded rectangular grid. Let $U=\{u(i)\,|\, i\in \Omega \}$ and $V=\{v(i)\,|\, i\in \Omega \}$ be the true and noisy images, respectively, such that

$$\begin{aligned} v(i)=u(i)+n(i),\quad i=(i_1,i_2)\in \Omega , \end{aligned}$$

where *u*(*i*) and $v(i)\in {\mathbb {R}}_+$ are the intensities of gray level and *n*(*i*) is an independent and identically different Gaussian random noise with zero mean and variance $\sigma ^2$ at pixel $i\in \Omega$. The continuous image is interpreted as the Shannon interpolation of the discrete grid of samples *v*(*i*) over $\Omega$. The goal here is to test the performance of method on *noisy imageV* in order to analyse the behaviour of proposed method in comparison with its test on the true image *U*. For this purpose three different noise levels with $\sigma = 25$, 50 and 75 are considered in Fig. 8 to test the significant application of the proposed algorithm. It can be observed that in case of noisy environment slight variations occur in visual quality and quantitative results as shown in Fig. 9 and Table 6. One can see that the entropy level of noise corrupted pixels is decreasing with increase in the level of Gaussian random noise. It shows most of the pixels are adopting similar grey levels in random data instead of particular arrangement of pixels in the original image. The contrast level also decreases with increasing noise level. Similarly changes in other parameters can be observed. The comparative analysis performed by applying AES S-box at the same noise levels is also shown in Table 7 and Fig. 10. One can observe that, with the increase in noise, the visual and numerical results obtained by the newly designed S-box are better or at least pretty similar to the recent state-of-the-art AES S-box (Daemen and Rijmen 2002).

Based on the experimental results regarding the overall performance of our proposed algorithm, it is demonstrated that the newly synthesized S-box satisfies all the criteria of acceptability to be used for secure communication.

## Conclusion

In this work we propose an S-box structured by an extremely simple and direct algorithm. Its strength is analyzed by several tests and it is self-evident that its confusion creating capability is quite high as compared to some other very famous S-boxes. The algebraic complexity based on the fractional linear transformation produces ideal results that make this S-box authentic and more reliable.

## References

[CR1] Abuelyman ES, Alsehibani AAS (2008). An optimized implementation of the S-box using residue of prime numbers. Int J Comput Sci Netw Secur.

[CR2] Bao L, Zhou Y (2015). Image encryption: generating visually meaningful encrypted images. Inf Sci.

[CR3] Benvenuto CJ (2012). Galois field in cryptography.

[CR4] Biham E, Shamir A (1991). Differential cryptanalysis of DES-like cryptosystems. J Cryptol.

[CR5] Carlet C, Ding C (2004). Highly nonlinear mappings. J Complex.

[CR6] Carlet C, Ding C (2007). Nonlinearity of S-boxes. Finite fields and their applications.

[CR7] Cui L, Cao Y (2007). A new S-box structure named Affine-Power-Affine. Int J Innov Comput I.

[CR8] Daemen J, Rijmen V (2002). The design of Rijndael-AES: the advanced encrytion standard.

[CR9] Detombe J, Tavares S (1992) Constructing large cryptographically strong S-boxes. In: Advances in cryptology-AUSCRYP’92. Lecture notes in computer science, vol 718, pp 165–181

[CR10] Gao T, Chen Z (2008). A new image encryption algorithm based on hyper-chaos. Phys Lett A.

[CR11] Hussain I, Shah T, Gondal MA, Khan M, Khan WA (2011). Construction of new S-box using linear fractiional transformation. World Appl Sci J.

[CR12] Hussain I, Shah T, Gondal MA, Mahmood H (2012). Generalized majority logic criterion to analyze the statistical strength of S-boxes. Z Naturforsch A.

[CR13] Hussain I, Shah T, Gondal MA, Khan WA, Mahmood H (2013). A group theoretic approach to construct cryptographically strong substitution boxes. Neural Comput Appl.

[CR14] Hussain I, Shah T, Mahmood H, Gondal MA (2013). A projective general linear group based algorithm for the construction of substitution box for block ciphers. Neural Comput Appl.

[CR15] Kellen D (2014) Implementation of bit-vector variables in a CP solver, with an application to the generation of cryptographic S-boxes. In: Masters Dissertation Uppsala Universitet

[CR16] Kim J, Phan RCW (2009). Advanced differential-style crypt-analysis of the NSA’s skipjack block cipher. Cryptologia.

[CR17] Matsui M (1998) Linear cryptanalysis method for DES cipher. In: Proceedings of EUROCRYPT’93. Springer, Berlin, pp 386–397

[CR18] Murguia JS, Flores-Erana G, Carlos MM, Rosu HC (2012). Matrix approach of an encryption system based on cellular automata and its numerical implementation. Int J Mod Phys.

[CR19] Nyberg K (1992) Perfect nonlinear S-boxes. In: Advances in cryptology-EUROCRYPT’91. Lecture notes in computer science, vol 457. Springer, pp 378–386

[CR20] Nyberg K (1993) On the construction of highly nonlinear permutations. In: Advances in cryptology-EUROCRYPT’92. Lecture notes in computer science, vol 658. Springer, Heidelberg, pp 92–98

[CR21] Ramamoorthy V, Silaghi MC, Matsui T, Hirayama K, Yokoo M (2011) The design of cryptographic S-boxes using CSPs. In: Principles and practice of constraint programming. Lecture notes in computer science, vol 6876. Springer, Berlin, pp 54–68

[CR22] Ramirez-Torres MT, Murguia JS, Carlos MM (2014). Image encryption with an improved cryptosystem based on a matrix approach. Int J Mod Phys.

[CR23] Shannon CE (1949). Communication theory of secrecy systems. Bell Syst Tech J.

[CR24] Shi XY, You XCXH, Lam KY (2002). A method for obtaining cryptographically strong $$8\times 8$$ S-boxes. Int Conf Inf Netw Appl.

[CR25] Tran MT, Bui DK, Doung AD (2008) Gray S-box for advanced encryption standard. In: International conference computational intelligence and security, pp 253–256

[CR26] Vargas-Olmos C, Murguia JS, Ramirez-Torres MT, Carlos MM, Rosu HC, Gonzalez Aguilar H (2015). Two-dimensional DFA scaling analysis applied to encrypted images. Int J Mod Phys.

[CR27] Vargas-Olmos C, Murguia JS, Ramirez-Torres MT, Carlos MM, Rosu HC, Gonzalez-Aguilar H (2016). Perceptual security of encrypted images based on wavelet scaling analysis. Phys A.

[CR28] Webster AF, Tavares SE (1986) On the design of s-boxes. In: Advances in cryptology: proceedings of CRYPTO’85. Springer, Berlin, pp 523–534

